# Nasal Septal Deviation: A Comprehensive Narrative Review

**DOI:** 10.7759/cureus.31317

**Published:** 2022-11-10

**Authors:** Fahad S Alghamdi, Dhai Albogami, Atheer S Alsurayhi, Anmar Y Alshibely, Tariq H Alkaabi, Laila M Alqurashi, Ali A Alahdal, Anfal A Saber, Omar S Almansouri

**Affiliations:** 1 Family Medicine, King Fahad Armed Forces Hospital, Jeddah, SAU; 2 College of Medicine, King Saud Bin Abdulaziz University for Health Sciences College of Medicine, Jeddah, SAU; 3 Otolaryngology - Head and Neck Surgery, King Saud University for Health Sciences, Jeddah, SAU; 4 Psychiatry, College of Medicine, King Saud University for Health Sciences, Jeddah, SAU; 5 General Practice, King Faisal Hospital, Makkah Almukarramah, SAU; 6 Otolaryngology - Head and Neck Surgery, College of Medicine, King Saud University for Health Sciences, Jeddah, SAU; 7 Forensic Medicine, Forensic Medicine Center, Makkah Almukarramah, SAU; 8 Family Medicine, College of Medicine, King Saud University for Health Sciences, Jeddah, SAU

**Keywords:** nasal deformity, quality of life (qol), septoplasty, cosmetic, nasal septum deviation

## Abstract

The nasal septum is an osteocartilaginous wall that divides the nose into two nasal cavities. Asymptomatic minor deviation of the septum is considered a normal developmental variation found in the majority of the population. The reported global prevalence rates had great variation due to the extent of deviation considered in the reporting studies. Previous classification systems have been proposed to classify the nasal septal deviation according to the characteristics of the nasal septum seen horizontally and vertically. For some patients, the degree of the deviation may affect the nasal airflow causing obstruction or impairing the olfactory function. Headache, rhinosinusitis, high blood pressure, obstructive sleep apnea, and breathing sounds are also among the clinical presentations of nasal septal deviation. Clinical assessment is sufficient to make the diagnosis while imaging techniques are required for decision-making. Radiological imaging techniques such as computed tomography (CT) are used to classify and assess the severity of the deviated septum. Surgical correction is the treatment option for nasal septal deviation. Septoplasty is the most common procedure used for nasal correction with high satisfaction levels and low complication rates. In this review, we present a comprehensive summary of the concept, presentation, diagnosis, management options, and quality of life of patients with nasal septal deviation.

## Introduction and background

The nasal septum is a complex osseocartilaginous structure that divides the nose into two nasal passages [1]. Generally, it is rare to have a symmetrical nasal cavity, and some degree of deviation is considered a normal anatomical variation [2]. However, nasal septum deviation (NSD) can be either developmental which is generally a smooth “C-shaped or S-shaped” deformity, or a result of trauma which is usually more dislocated and irregular [1].

**Figure 1:**
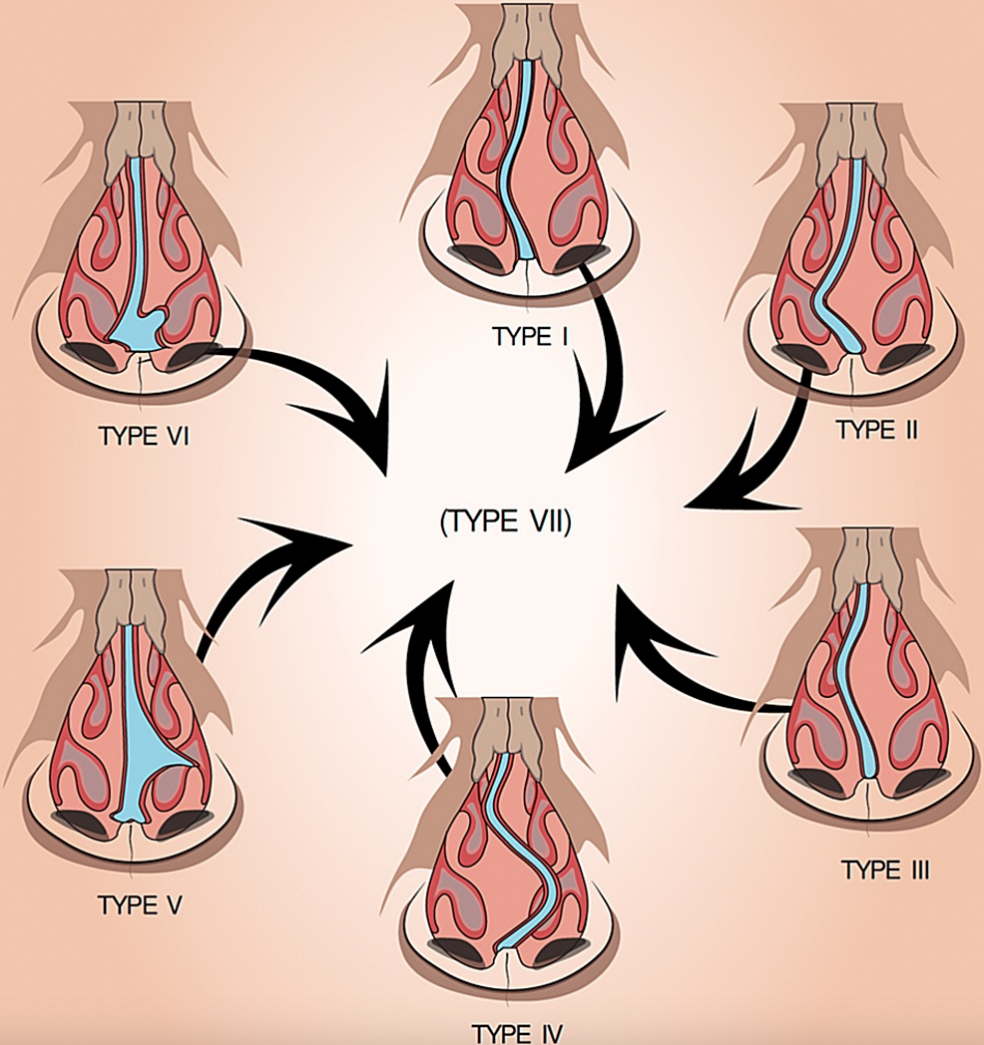
Mladina’s classification of nasal septal deviation (NSD) Type VII is a combination of more than one of the six types. The graph is a visual demonstration of the physical concepts of Mladina’s classification created by the authors of this article

The wide variation of NSD structure, symptoms and associated comorbidities has evolved the development of classification systems. NSD can be classified according to extent of the nasal deviation on the inferior turbinate [3]. This classification has three degrees, degree I comprises a septal deviation without reaching the inferior turbinate, degree II represents a deviation reaching the inferior turbinate, and degree III involves a septal deviation reaching and compressing the inferior turbinate [3]. Another classification relies on the commonly noticed deviation patterns such as S-shaped and C-shaped deviations [4]. Mladina’s classification system has been proposed to classify the NSD according to the characteristics of the nasal septum seen horizontally and vertically on rhinoscopy or cone-beam computed tomography (CBCT) [5]. Mladina’s system classifies nasal septum deviation into seven types; type I involves the vertical ridge without reaching the nasal dorsum; type II involves the vertical ridge reaching the nasal dorsum; type III involves the vertical ridge in a deeper area; type IV involves the anterior and the deeper areas of the vertical ridge; type V manifests as a horizontal deformity on one side of the nose with the other being flat; type VI manifests a bilateral involvement of the septum with dislocation of one side and deviation of the other side; type VII represent as a combination of two or more types [5,6]. See the approximate demonstration of Mladina’s classification in Figure 1.

More accurate diagnostic techniques such as CBCT revealed higher prevalence rates of NSD reaching 86.6%. Higher prevalence rates were found among older age groups while no associations were found with gender and history of trauma. The relation with older age can suggest a developmental influence on NSD as type VII was the most common type found [7]. This was similar to the reported prevalence in the United Arab Emirates of 74.5% [8]. Globally, there is a high variation in the reported prevalence rates of NSD ranging from 26% to 97%, which can be explained by the extent of deviation considered in the reporting studies [9]. Among neonates in India, the prevalence of nasal septal deviation was found to be 20% diagnosed using clinical examination by Gray’s struts among those aged “2” days. Higher numbers were observed among primiparas and instrumental deliveries [10].

Management of NSD is either surgical or non-surgical. The choice of intervention usually depends on the main complaint. For example, the management of allergic rhinitis is by the long-term use of steroid nasal spray. Nevertheless, septal surgery is superior and most effective than nonsurgical interventions [11]. Septoplasty is favorable in patients with obstructive symptoms, with a satisfaction rate ranging from 50% to 100% [12].

## Review

Associated conditions

In a study that investigated the relationship between NSD and rhinosinusitis, 50% of the patients with rhinosinusitis had NSD. While the relationship was not statistically significant, types I, II and V were more prone to have rhinosinusitis. Type I and type II are thought to be associated with rhinosinusitis due to the involvement of the nasal valve area [4]. Another study reported a significant association between bilateral maxillary sinusitis and NSD [13]. The severity of NSD was significantly associated with the presence of rhinosinusitis among patients undergoing endoscopic sinus surgery compared to asymptomatic controls. However, the morphological type of NSD had no association with the presence of NSD [14].

Concha bullosa (CB) is an anatomical variation of the sinonasal structure caused by pneumatization of the nasal turbinate. While the exact cause of CB is still not known, previous studies have tried to explore the relationship between CB and NSD which revealed that the frequency of CB was higher among patients suffering from NSD without a clear etiological explanation. However, the angle of the deviation positively influences the size of the CB [15]. In contrast, while not statistically significant, mild cases of NSD had higher rates of CB compared to patients with severe NSD [8]. The side of the septal deformity was also found to affect the incidence of CB in the contralateral direction of NSD [13].

The incidence of headaches was found to be higher among patients with NSD. This finding was suggested after a follow-up period of 10 years and adjustment for sex, age and socioeconomic status [16]. This can be explained by the fact that patients with NSD are more prone to oxidative stress [17]. While no etiological association could be drawn between NSD and antral pseudocysts, the prevalence was found to be higher among those with NSD [18]. In a study among Canadian otolaryngologists, concerns reported included the low accuracy of physical examination to decide the need for functional surgical correction for NSD [19]. The displacement caused by the deviation leads to the narrowing of the contralateral nasal cavity which eventually affects the airflow and may cause obstruction in severe cases. Furthermore, septal deviation interferes with surgical procedures [20]. The presence of air cells in the posterior part of the septum attached to the sphenoid sinus can block drainage or pass infection among patients with NSD [20].

Anatomy

The nasal septum is in the middle of the nasal cavity, dividing it into left and right cavities. Specifically, the nasal septum forms the medial wall of the nasal cavity. The anterior side of the nasal cavity is made of septal cartilage while the posterior part is composed of a vomer and perpendicular plate of the ethmoid. The nasal cavity is also inferior to the cribriform plate of the ethmoid bone. The floor of the nasal cavity is made by the articulation of the palatine process of the maxilla anteriorly, and the palatine bone posteriorly forming the hard palate. The lateral wall of the nasal cavity has nasal conchae or turbinates which are divided into three parts, superior, middle and inferior which allow for humidification, warming and filtration of the inspired air [21].

The variation in the anatomy of the nasal cavity may influence the airflow with little effect on warming function. The variations were evident across the different types of nasal deviation [22]. Therefore, having a severe deviation in the nasal septum will disturb the normal process of respiration leading to nasal obstruction, sinusitis, and snoring [23].

Clinical evaluation and diagnosis 

The asymptomatic presence of the deviated septum is the typical case in the affected population. Past history may reveal a trauma, while the association with the mode of delivery is still controversial [24]. It is also important to investigate patients with recurrent unexplained epistaxis for nasal deviation and nasal obstruction [25].

The presence of headache as a presenting symptom for some patients was explained by the contact between the convex side and the mucosa of the peripheral nasal wall of the inferior turbinate or middle turbinate, or the lateral nasal wall of the nasal septum [16]. The end of the sensory nerve is located between them, and it will be affected and cause pain. It is reported that in such rhinogenic headaches, surgical treatment is better than medical treatment [16]. The same study suggested septoplasty as one of the treatment options for unexplained headaches [16].

The presence of nasal sounds is usually found among those who are suffering from obstruction or those having narrow nasal cavities which correlates with the severity of the deviation. This highlights the beneficiary utilization of acoustic rhinometry (AR) in the diagnosis of anterio-caudal deviation. This indeed does not exclude the importance of endoscopic examination prior to septoplasty [26]. Clinical evaluation as opposed to imaging has both been shown to be underestimating some types of NSD, both options should be sought before decision-making in clinical settings [27].

Moreover, nasal obstruction can be caused by septal deviation which can lead to obstructive sleep apnea (OSA), which is a sleep disorder that involves cessation or significant decrease in airflow in the presence of breathing effort [28]. In fact, the prevalence of OSA was 4.39 times higher in the NSD group compared with the control group. Septoplasty, therefore, is considered one of the solutions to decrease OSA significantly, especially in patients with increased BMI [29]. However, other conditions to be sought when assessing a patient with NSD include rhinosinusitis, CB, and chronic otitis media [30]. The diagnosis of NSD can be made through different clinical tests. For example, the position and degree of nasal septal deviations can be determined by anterior rhinoscopy and nasal endoscopy when done in a decongested situation, but it is a distressing procedure that has a high inter-rater range [31].

AR measures the acoustic reflection of a sound signal by structures within the nasal cavity to evaluate nasal patency. Moreover, rhinomanometry (RMM) offers a dynamic physiologic evaluation of the nose, it calculates nasal resistance by detecting intranasal pressure and nasal volume flows to measure nasal ventilation. Nasal sound spectral analysis (NSSA) could provide an indirect approach to monitoring nasal airflow dynamically by calculating nasal cavity noise induced by turbulent nasal airflow It is very simple and affordable to carry out [31].

CBCT is considered a reliable radiological option for the evaluation of nasal obstruction and NSD. Different parameters such as septal deviation angle and septal deviation index can be computed from the CBCT that were found to be significantly different among patients with NSD [32]. For pre-operative assessment, nasal endoscopy and craniofacial CT are more accurate and precise than nasal pyeloscopy and “3D” reconstruction [33]. Variations in the surgical decision-making for patients with NSD can be influenced based on the judgment solely made on clinical examination or radiological imaging. The majority of cases experience a modification in the surgical plan after radiological imaging [34].

Surgical options

The most common surgical method used to treat NSD in adults is septoplasty [11]. Septoplasty is a common otolaryngological surgical procedure, which involves the correction of a deviated septum, expanding the nasal passage and allowing adequate airflow [11,35]. Indications for septoplasty include septal deviation with symptomatic obstruction, gaining access for endoscopic sinus surgery and lead point headaches [35]. Septoplasty is usually an open procedure done when dealing with a caudal septal deviation but when there is a posterior septal deviation surgeons prefer to use endoscopic septoplasty due to its advantage of providing the surgeon with a better visualization compared to open septoplasty. However, choosing one of these techniques depends on the capabilities, skills, and preferences of the surgeon [36]. According to a study that was made on 141 individuals who suffered from a caudal septal deviation to assess the outcome of septoplasty procedure using bony batten grafting showed a good result with a favorable outcome [37]. In severe cases of NSD, extracorporeal septoplasty is recommended. It can be an open or closed procedure in which the whole (cartilaginous and bony) septum is extracted and reconstructed. This procedure was reported to give an optimal functional improvement [38].

The selection of the appropriate technique in septoplasty can vary across the patients according to the type of nasal deviation. For antero-caudal septal deviation, extracorporeal septal reconstruction was found to be more effective than endonasal septoplasty [39]. However, the operation should be corrected as soon as possible for children to enhance better outcomes and conserve the cartilage [40]. Fashioned mucoperichondrium flap has been satisfactory for patients with symptomatic caudal septal deviation [41]. Rhinoplasty procedure commonly done with septoplasty and called rhinoseptoplasty which is used to treat and relieve symptoms of obstruction and improve breathing. Rhinoplasty may be considered as a secondary option for treating nasal cartilage deviation along with a cosmetic or aesthetic purpose [42]. Septal deviation can also be accompanied by turbinate hypertrophy; thus, septoplasty may be performed concurrently with turbinate surgery [11]. Nasal symptoms in septoplasty patients can also be assessed using the Nasal Obstruction Septoplasty Effectiveness (NOSE) and the Sinonasal Outcome Test-20 (SNOT-20) questionnaire scale [43].

Rapid maxillary expansion (RME) has also been shown to improve nasal ventilation among children. While not statistically significant, RME was reported to increase the volume of the nasal cavity, nasopharynx, and oropharynx [44]. Among symptomatic patients with NSD without nasal deformity, we aim to preserve the cartilage and improve nasal functions. A suitable technique that should be used for patients without external deformity has been proposed by Zhao et al., with rare complications [45]. The availability of uncomplicated techniques provides a safe option for the patients and expands the indications for physicians. A good prognosis was also reported to improve the quality of life significantly as 70.6% of the patients indicate improved smell after surgical correction of NSD [46].

For the patient, the purpose to undergo a septoplasty is to improve nasal and olfactory functions or as a cosmetic procedure. Alongside the patient’s desire, physicians should also be encouraged to perform the surgery to improve cardiovascular function as well. Patients’ satisfaction has been measured by the smell and respiratory perspective. Cardiovascular effects of septoplasty have been reported as well. In a comparison between pre-and post-surgery, patients who underwent septoplasty, systolic blood pressure was reduced after septoplasty [47]. This case scenario often comes among patients less than 35 years of age and having idiopathic hypertension [48].

The reported most common complications include bleeding, cerebrospinal fluid (CSF) rhinorrhea, extraocular muscle damage, septal perforation, sensory changes, saddle nose deformity, nasal tip depression, infection, septal abscess, and toxic shock complications that may result from this procedure [49]. Another study found that excessive bleeding was the most common consequence [50], while another study found that deformities, infections, and perforations are the most frequent side effects after septoplasty [51]. Moreover, bacteremia may occur post-operatively which is more common in those who bled more during the procedure [52]. In addition, another study found that postoperative infection (3.3%) and intervention-required epistaxis (4.5%) were the only short-term problems. However, of the total, 2.8% experienced long-term problems, including hyposmia and subsequent septoplasty [53].

Patient's quality of life

Nasal obstruction was found to affect the patient's quality of life (QOL) significantly, and recovery of the olfactory nerve has positively influenced the psychological status of patients with nasal obstruction [54]. The improvement in psychological status was linked to the clinical improvement of nasal functions [54]. In addition to reduced QOL, the rates of depression and anxiety were found to be higher among patients with NSD, which should encourage psychological distress counseling [55].

The decrease in QOL is mainly obstruction-related, as NSD did not affect the QOL for inferior turbinate hypertrophy patients [56]. Nasal obstruction is thought to affect sleep quality along with psychiatric symptoms such as somatization, obsession, hostility and interpersonal sensitivity due to reduced physical health [57]. Despite the low likelihood of septal deviation as a major cause for decreased QOL, the improvement of QOL among children was reported [58].

Patient symptoms such as headache and facial pain can highly impact the quality of life and daily activities. Multiple clinic checkups to be cleared to visit an otorhinolaryngology (ORL) clinic, especially in the absence of an active nasal inflammatory condition may further impact the psychological profile of the patients [59].

To further evaluate the psychological profile of patients a study was done to evaluate index of quality of life by analyzing patients with nasal septal deviation before and after the surgery, using the self-rating anxiety scale (SAS) and self-rating depression scale (SDS), and the results of that study showed that the preoperative scores of SAS/SDS for patients with nasal septal deviation are higher than those of the national standards and the level of anxiety and depression are lower than the national norm postoperatively and the patient with normal to moderate SAS/SDS scores are better postoperatively but the patient with severe SAS/SDS scores had poor improvement of their symptoms [60].

## Conclusions

The aim of this article was to provide a comprehensive review regarding nasal septum deviation, surgery indications and techniques. The normal anatomical variation in the nasal septum may pass through life with no symptoms. In the presence of obstruction or symptoms related to septal deviation, septoplasty can be indicated. Septoplasty is considered the most suitable option to correct NSD. The selection of the appropriate technique depends on the type of deviation and individual characteristics. The surgery while considered convenient with greater benefits, incidental negative side effects may occur. Post-operative complications such as bleeding and deformity are rare and surgical interventions have shown a great improvement in the quality of life of the patients with high levels of satisfaction among the patients. Other post-operative positive effects such as lowered systolic blood pressure have been reported as a desired cardiovascular side effect after the correction of NSD. The clinical decision of the surgery should be made after careful examination and CBCT while accounting for individual patients’ risks.
